# Several Genes Encoding Enzymes with the Same Activity Are Necessary for Aerobic Fungal Degradation of Cellulose in Nature

**DOI:** 10.1371/journal.pone.0114138

**Published:** 2014-12-02

**Authors:** Peter K. Busk, Mette Lange, Bo Pilgaard, Lene Lange

**Affiliations:** Department of Biotechnology, Chemistry and Environmental Engineering, Aalborg University, A.C. Meyers Vænge 15, 2450, Copenhagen SV, Denmark; Consejo Superior de Investigaciones Cientificas, Spain

## Abstract

The cellulose-degrading fungal enzymes are glycoside hydrolases of the GH families and lytic polysaccharide monooxygenases. The entanglement of glycoside hydrolase families and functions makes it difficult to predict the enzymatic activity of glycoside hydrolases based on their sequence. In the present study we further developed the method Peptide Pattern Recognition to an automatic approach not only to find all genes encoding glycoside hydrolases and lytic polysaccharide monooxygenases in fungal genomes but also to predict the function of the genes. The functional annotation is an important feature as it provides a direct route to predict function from primary sequence. Furthermore, we used Peptide Pattern Recognition to compare the cellulose-degrading enzyme activities encoded by 39 fungal genomes. The results indicated that cellobiohydrolases and AA9 lytic polysaccharide monooxygenases are hallmarks of cellulose-degrading fungi except brown rot fungi. Furthermore, a high number of AA9, endocellulase and β-glucosidase genes were identified, not in what are known to be the strongest, specialized lignocellulose degraders but in saprophytic fungi that can use a wide variety of substrates whereas only few of these genes were found in fungi that have a limited number of natural, lignocellulotic substrates. This correlation suggests that enzymes with different properties are necessary for degradation of cellulose in different complex substrates. Interestingly, clustering of the fungi based on their predicted enzymes indicated that *Ascomycota* and *Basidiomycota* use the same enzymatic activities to degrade plant cell walls.

## Introduction

Since fungi evolved enzymes for degradation of plant cell wall material they have been important contributors to the natural turnover of plant cell wall material [1]. Hence, fungi have the capacity to degrade both the polysaccharides and the lignin that are the major components of plant cell walls [2]–[5].

Cellulose is the simplest of the three most abundant polysaccharides in plant cell walls, cellulose, hemicellulose and pectin. Cellulose consists of linear structures of β-1,4-linked D-glucose that can be degraded to oligomers by the combined action of cellobiohydrolases, endocellulases and lytic polysaccharide monooxygenases (LPMO) and further degraded to glucose by β-glucosidases [2], [6], [7]. However, in its natural context the cellulose is protected from degradation by the complex structures of plant cell walls including both covalent links between cellulose and hemicellulose and entanglement of the cellulose with other macromolecules [8]. The presence of these structures turns enzymatic degradation of cellulose in nature into a complicated task that depends on degradation of the other components of the plant cell wall to facilitate the access of cellulose-degrading enzymes to their substrate [9]. For example, several reports show that the presence of hemicellulose-degrading enzymes can seriously enhance enzymatic hydrolysis of cellulose [10]–[13].

The CAZy database (www.cazy.org) classifies the enzymes degrading cellulose and other polysaccharides of the plant cell wall into the glycoside hydrolase (GH) families [14]. The GH families are based on protein sequence and structure and do not necessarily reflect activity. Thus, most of the families comprise proteins with different enzymatic activities and proteins with the same enzymatic activity can be found in different GH families. One example is that endoglucanases (EC 3.2.1.4) belong to 17 different GH families and one of these families, GH5, includes 20 different enzymatic activities. The GH5 family has been divided into functionally-relevant subfamilies but this work required a tremendous effort including manual curation of the subfamilies [15]. Thus, the division into subfamilies has only been done for GH5 and a few others of the CAZy families.

Like the endoglucanases, the other glycoside hydrolases that are directly involved in cellulose-decomposition can be found in several GH families. Β-glucosidases are found in six GH families and each of the cellobiohydrolases distributed in three GH families (www.cazy.org). Moreover, each of these GH families includes proteins with between 2 and 22 different activities. Hence, annotation of a gene to the GH3 family does not necessarily mean that the gene encodes a β-glucosidase as 10 different functions have been described for the GH3 proteins.

The GH family 61 was shown to include fungal LPMOs [16]–[19] that were later reclassified as auxiliary activities with the AA9 family containing all known fungal cellulase-acting LPMOs [20] and the AA11 family containing the only described fungal chitin-acting LPMO [21]. The AA11 proteins share several structural characteristics with the AA9 proteins and some of them were originally described as GH61 proteins [22]. Hence, it cannot be excluded that the AA11 family includes both chitin- and cellulose-acting enzymes like it has been found for the bacterial LPMOs in the AA10 family [23], [24].

Due to the entanglement of glycoside hydrolase families and functions it is not an easy task to predict the enzymatic activity of glycoside hydrolases based on their sequence [14], [25]. Hence, comparative analysis of the genomes of polysaccharide-degrading fungi has been based on the number of genes encoding members of each glycoside hydrolase family as an indirect measure of the cell wall-degrading capacity of the fungi [1], [26], [27]. Although the enzymatic activity of each gene was not predicted, these studies demonstrate that different fungi have different enzyme types that reflect different approaches to plant cell wall degradation.

Peptide Pattern Recognition (PPR) is a new approach for sequence analysis [22]. This method was used to classify glycoside hydrolases of the GH5 and GH13 families into subfamilies that correlate largely with the enzymatic function of the proteins. Thus, PPR can be used to predict the function of known GH-encoding genes.

Here we used PPR to classify GH families 1–131 and the AA families 9–11 (i.e. the LPMOs) into subfamilies and showed that the subfamilies could be used not only to predict the function of known GH-encoding genes but also to find all GH- and LPMO-encoding genes in a fungal genome and predict their functions. This method was used to mine the genomes of cellulose-degrading and non-degrading fungi [1]–[5], [28] for all GH- and LPMO-encoding genes and to predict the function of the encoded enzymes. The results points to that *Ascomycota* and *Basidiomycota* acquired the same enzymatic activities for cellulose-degradation after their divergence into different fungal divisions. Moreover, a high number of genes encoding endo-glucanases, β-glucosidases and LPMOs were found in most of the cellulose-degrading fungi in agreement with the hypothesis that for each of these three enzyme-activities several versions with different properties are necessary for the decomposition of cellulose in natural plant-cell wall substrates.

## Materials and Methods

### Genomes

Genome sequences of the fungi were either downloaded from public databases or obtained by sequencing of genomic DNA as indicated (Table S1).

For *de novo* sequencing, DNA was extracted as described [29] and sequenced on an Illumina Hiseq 2000 in one multiplexed lane as paired-end libraries with Truseq chemistry by AROS Applied Biotechnology A/S, Denmark. Based on estimated genome sizes the calculated sequence coverages ranged between 130x–230x for the genomes.

Before assembly the raw sequences were filtered for residual adapter sequences and trimmed using AdapterRemoval v1.5.2 [30] and Seqtk [31] and error corrected with Quake v0.3 [32]. Redundant reads were removed through digital normalization with Diginorm in Khmer v0.5 package [33].

The genomes were assembled to scaffolds with Velvet v1.2.10 [34] or CLC Genomic Workbench (CLC Bio, Denmark). Several k-mer sizes and coverage values were tested to obtain the longest possible contigs with the lowest gap occurrence (Table S2).

Sequence data have been deposited in NIH Short Read Archive as part of the Bioprojects PRJNA261108, PRJNA261109, PRJNA261270 and PRJNA261275.

All contigs shorter than 500 base pairs were discarded before further analysis.

### Peptide Pattern Analysis

Amino acid sequences and information about enzymatic activity (EC numbers) of all GH and of AA9 and AA10 proteins were downloaded from CAZy (www.cazy.org [35]) in June 2013. AA11 [21] was downloaded in January, 2014 and pooled with the GH61 subfamily 7 [22], which includes the same type of proteins as AA11. Each protein family was analyzed by PPR. Briefly, for each protein family PPR found the largest group of proteins that contained at least 10 of 70 conserved hexamer peptides as previously described for the GH13 and GH61 protein families [22]. The length of the conserved peptides (hexamers), the number of conserved peptides per protein (10) and the total number of conserved peptides per group (70) were chosen as they were the conditions that gave the best rate of prediction of protein function in empirical testing of peptide lengths from trimers to decamers, 5–40 conserved peptides per protein and 30–200 conserved peptides per group [22]. Moreover, a conserved peptide was defined as a peptide that is found in at least 20% of the proteins in a group as this definition was used for finding the PPR parameters giving the best rate of prediction of protein function [22]. This definition implies that any peptide found in less than 20% of the proteins in a group of proteins will be removed from the list of conserved peptides. For example, if peptide number 70 is found in only 15% of the proteins it will be removed and the list of conserved peptides will only contain 69 peptides.

The first group of proteins identified by this method was defined as subfamily 1. Next, PPR found the second largest group of proteins, not including any proteins from subfamily 1, defined by the same criteria. This group of proteins was defined as subfamily 2 and so on. PPR continued the analysis until less than five proteins could be grouped in this way. All GH subfamilies containing proteins with a reported enzymatic activity described in CAZy were assigned the same function as the proteins with a reported enzymatic activity as previously described [22]. All AA9, AA10 and AA11 proteins were assumed to be LPMOs.

### Gene annotation by finding homology to peptide patterns (Hotpep)

**Figure 1:**
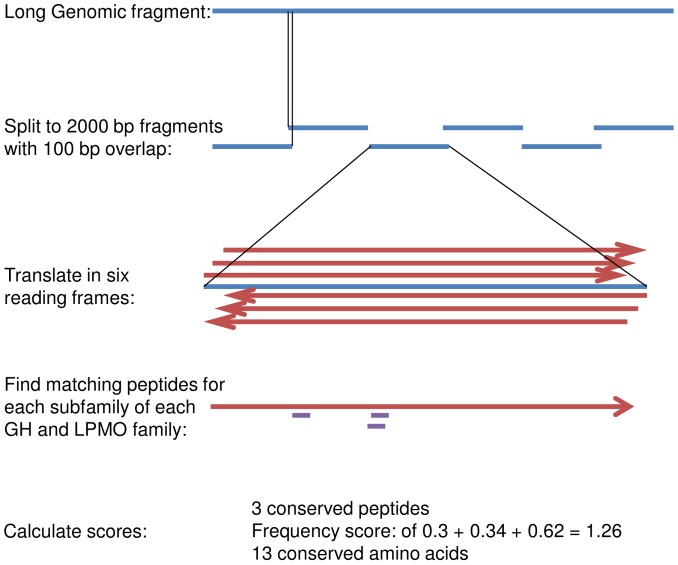
Flow scheme for the Hotpep analysis. Blue lines represent DNA sequences. Red arrows represent protein sequences. Short purple lines represent short peptides. See “Materials and Methods” for details.

The genomes were split into 2000 bases long fragments with 100 bases overlap between fragments (Fig. 1). Each fragment was translated in all six reading frames, which was given a score for each subfamily-specific peptide lists for each GH and AA family by:

1. Finding all the peptides from the list that were present in the reading frame.

Example: A protein sequence may contain the three conserved peptides FGTFHL, GTFHLY and YIKSLD (single-letter amino acid code).

1. Sum the frequency of these peptides. This gave the subfamily-specific frequency score.

Example: If the peptide FGTFHL is found in 30% of the proteins in the family, it has a frequency of 0.3. Likewise GTFHLY has a frequency of 0.34 if it is found in 34% of the proteins in the family and the frequency of YIKSLD is 0.62 if it is found in 62% of the proteins in the family. Thus, a protein containing these three peptides has a subfamily-specific frequency score of 0.3+0.34+0.62 = 1.26.

 A hit was considered significant if one of the open reading frames:

1. Included at least three conserved peptides from a subfamily.
2. The sum of the frequency of these peptides was higher than 1.0
3. The conserved peptides covered at least ten amino acids of the ORF. (Three hexapeptides that may be overlapping can cover from 8 (maximal overlap) to 18 (no overlap) residues of an amino acid sequence).

Example: A protein sequence contains the conserved peptide FGTFHL at residues 110–115, the conserved peptide GTFHLY at residues 111–116 and YIKSLD at residues 64–69. As the peptides FGTFHL and GTFHLY share five amino acids they cover a total of seven amino acids (residues 110–116). Together with the six amino acids covered by YIKSLD it gives a total of 7+6 = 13 conserved amino acid residues.

If all three conditions were met a sequence fragment was assigned to the GH or AA family and to the PPR subfamily with the highest subfamily-specific frequency score as previously described [22]. If two fragments were assigned to the same GH or AA family and the distance between them in the original genome sequence was less than 5800 bases, the fragments were considered to be part of the same gene and counted as one hit.

Finally, if a function had been assigned to the subfamily the hit was assigned the same function [22].

Although each sequence fragment could only be assigned once to each GH or AA family it was possible for a fragment to be assigned to two or more different families thus taking the modular structure of CAZymes into account [20], [36].

### Genome and sequence comparisons

R package version 3.0.1 was used for Ward hierarchical clustering. BLAST search [37] for sequence alignment was done by standard methods and conserved domains were identified in the CDD database at NCBI [38].

### Statistical analysis

Student's T-test was used for comparisons of data sets as indicated.

## Results

### Description of Hotpep analysis

The conserved peptides identified by PPR are useful for design of degenerate primers for PCR amplification of related genes [22]. Moreover, by searching for homology to peptide patterns (method named Hotpep hereafter), it can be investigated whether a protein sequence contains a sufficient number of conserved peptides from a specific PPR-generated subfamily to be regarded as a member of this subfamily. Furthermore, if a sufficient number of the proteins used to generate the PPR subfamily have been characterized experimentally and have the same function, the new protein can be predicted to have the same function. This method for predicting protein function can be regarded as a kind of *in silico* PCR with degenerated primers and has been shown to correctly predict the function of approximately 80–90% of the proteins in selected GH families [22].

To be able to find all GH and LPMO encoding genes in a genome we performed a PPR analysis of all the proteins in the GH1 – GH131, AA9, AA10 and AA11 families in CAZy. This generated a number of subfamilies and subfamily-specific peptide lists for each family. Based on this analysis, any protein sequence that contains a sufficient number of peptides for one of these lists can be assigned to the specific subfamily of the particular CAZy family.

We wished to extend this method to identification of all the GH- and LPMO-encoding genes in a given fungal genome. To find all open reading frames in a genome of interest the contigs of the genome was divided into 2000 nucleotides long fragments with 100 nucleotides overlap. Next, each fragment was translated in all six reading frames. For each reading frame, the number of conserved peptides from each subfamily of all CAZy families was determined (Fig. 1). Each hit was characterized by three values: 1. The number of conserved peptides it contained. 2. The sum of the frequency of these peptides in the subfamily. 3. The number of amino acid residues covered by the peptides. Three hexapeptides that may be overlapping can cover from 8 (maximal overlap) to 18 (no overlap) residues of an amino acid sequence.

A protein sequence with few conserved peptides, all with low frequency and covering only a few amino acids of the protein will have a higher risk of being a false positive than a protein sequence with many conserved peptides with high frequency and covering many amino acids of the protein. Hence, different values for the three parameters were tested to empirically establish the threshold values that give the best balance between excluding false positives and avoiding false negatives. The classification of the predicted genes at specific threshold values was evaluated by comparison to the classification found in the CAZy database and by BLAST search (see below). This empirical testing showed that the optimal threshold values were: 1. A protein sequence should contain at least three conserved peptides. 2. The sum of the frequency of these peptides should be higher than 1.0. 3. The conserved peptides should cover at least ten amino acids of the ORF.

Any reading frame that satisfied these threshold conditions was considered a hit. If a reading frame contained a significant number of peptides from several subfamilies of the same CAZy family, the reading frame was assigned to the subfamily with the highest score. Although a reading frame was only assigned to one subfamily of each CAZy family no comparison of scores was made between families and hence, a sequence could be assigned to several families thus reflecting the modular nature of CAZymes [20], [36]. Hits in the same CAZy family found within a distance of 5800 nucleotides (on neighboring fragments or on fragments with one fragment in between) were assumed to belong to the same gene and counted as one hit.

Finally, each hit was assigned the same function as other proteins belonging to the same subfamily as previously described [22].

### Comparison between Hotpep and CAZy analysis of six genomes

To determine the efficiency of Hotpep for annotation of CAZymes and to test the empirically chosen threshold values we compared the GH and LPMO genes found by Hotpep to the genes annotated in the CAZy database (www.cazy.org) for six fungal genomes.

Hotpep found the same number of genes encoding proteins of each GH family and LPMOs (*P*>0.05; paired Student's T-test, Bonferoni corrected) for all six genomes (Table 1 and Table S3). On average, Hotpep found fewer genes (96%) in the six fungal genomes than the number annotated in CAZy (Table 1) but the difference was not significant (*P*>0.05; paired Student's T-test).

**Table 1:** CAZymes in fungal genomes found by PPR and annotated in CAZy.

Species	PPR genes	CAZy genes	Ratio (PPR/CAZy)
*S. cerevisia*	44	42	1.05
*A. nidulans*	254	260	0.98
*M. thermophila*	203	216	0.94
*T. terrestris*	214	227	0.94
*M. grisea*	241	251	0.96
*N. crassa*	187	186	1.01
Total	1143	1182	0.97

In total, hits were found in 68 GH and LPMO families in the six genomes. For individual families, significant differences (*P*<0.05, paired Student's T-test) between the number of hits found by Hotpep and reported in the CAZy database was limited to GH43 (*P* = 0.004) and GH128 (*P* = 0.033) (Table S4). However, when testing 68 families it is expected that 3.4 families will show a significant difference (*P*<0.05) because the statistical testing was done without correction for multiple comparisons (e.g. by Bonferoni correction). Hence, the difference between Hotpep and CAZy in the ability to predict GH-encoding genes was smaller than expected for *P*<0.05.

Nevertheless, to investigate putative systematic differences between the two methods we aligned the sequences of the 52 GH43 genes found by Hotpep to the 63 GH43 genes annotated by CAZy. The result showed that 52 of the CAZy-annotated GH43s matched 50 of the Hotpep hits (Table 2). In two instances did two CAZy-annotated GH43s match to the same Hotpep hit. This is explained by the fact that CAZy annotates domains and not genes per se and CAZymes can have several domains [36]. Hence, a gene with two GH43 domains will be counted twice by CAZy but only once by Hotpep. Of the 11 unmatched CAZy-annotated GH43s eight could be found by Hotpep if their full-length protein sequence was used for screening instead of using only the open reading frames of the fragmented genome as described in “Materials and Methods”. Three CAZy-annotated GH43s were not found by Hotpep and two GH43s found by Hotpep were not annotated in the CAZy genome database (Table 2).

**Table 2:** Comparison of GH43 genes found by PPR and annotated in CAZy.

CAZy annotated genes found by:	*A. nidulans*	*M. Thermophila*	*T. Terrestris*	*M. Grisea*	*N. Crassa*	Total
PPR in genomes	11^a^	11	8	17^a^	5	52
PPR in full-length protein sequences	14	12	9	18^a^	7	60
Not found by PPR	1	1	0	1	0	3
Found by PPR but not annotated in CAZy	2	0	0	0	0	2
All domains found by PPR	15	12	9	17	7	60
All domains annotated in CAZy	14	13	9	18	7	61

a Two CAZy hits to one PPR hit.

When accounting for the multidomain structure of the CAZymes and that some of the GH43 could only be found by Hotpep when they were properly assembled, Hotpep found 60 GH43 domains. This result was similar (*P* = 0.62; paired Student's T-test) to the 61 GH43 domains annotated in CAZy (Table 2).

### Selection of fungi

To pinpoint the GH and LPMO genes that are involved in cellulose degradation we selected a number of fungal genomes based on the ecological and physiological life-style and the taxonomy of the fungi (Table 3). The life-styles were selected to include fungi that have been described as degraders of cellulose in plant cell wall material containing other components such as hemicellulose and lignin [4], [5] instead of just adhering to a wider designation of cellulolytic fungi as fungi able to degrade any cellulose-containing material such as paper [39]. Hence, brown and white rot *Basidiomycota* and saprophytic ascomycetes able to live on cell walls from dead plants are designated as cellulose-degraders [1], [4], [5], [28], [40]. However, the *Zygomycota* of the *Mucorales* order, *Thermomucor indicae-seudaticae* and *Rhizopus delemar*, were classified as non-degraders based on their classification as fungi growing on easily accessible substrates [41] although they have a limited capacity for cellulose-degradation [42], [43]. On the other hand the dry rot fungus *Serpula lacrymans* was classified as a cellulose-degrader [44] despite a somewhat deficient repertoire of lignocellulose-degrading enzymes [45]. Overall, the cellulose-degraders included fungi that use a broad range of different strategies for biomass decomposition [1]–[3] and the selected species included fungi from four different divisions with cellulose-degraders and non-degraders from *Ascomycota* and *Basidiomycota* and non-degraders from *Chytridiomycota and Zygomycota* [4], [5], [28], [40].

**Table 3:** List of fungi whose genomes were mined for GH- and LPMO-encoding genes.

Fungus	Division	Description	Cellulose degrader
*Arthroderma gypseum*	Asco	Soil and skin	No
*Arthroderma otae*	Asco	Soil and skin	No
*Uncinocarpus reesii*	Asco	Environmental saprophyte	No
*Coccidioides immitis*	Asco	Soil-borne pathogen	No
*Saccharomyces cerevisiae*	Asco	Baker's yeast	No
*Wickerhamomyces anomalus*	Asco	Plants, animals, waste water	No
*Candida albicans*	Asco	Gut flora, pathogen	No
*Ceratocystis fimbriata*	Asco	Plant pathogen	No
*Laccaria bicolor*	Basidio	Ectomycorrhizal and saprobic	No
*Ustilago maydis*	Basidio	Plant pathogen	No
*Cryptococcus neoformans*	Basidio	Soil-borne pathogen	No
*Spizellomyces punctatus*	Chytridio	Soil	No
*Batrachochytrium dendrobatidis*	Chytridio	Amphibians	No
*Homoloaphlyctis polyrhiza*	Chytridio	Leaf litter	No
*Allomyces macrogynus*	Chytridio	Humid soil	No
*Rhizopus delemar*	Zygo	dead organic matter/pathogen	No
*Thermomucor indicae-seudaticae*	Zygo	Municipal compost	No
*Aspergillus nidulans*	Asco	Saprophyte	Yes
*Talaromyces stipitatus*	Asco	Saprophyte	Yes
*Myceliophthora thermophila*	Asco	Saprophyte	Yes
*Neurospora crassa*	Asco	Saprophyte	Yes
*Magnaporthe oryzae*	Asco	Cell-wall degrading plant pathogen	Yes
*Ascocoryne sarcoides*	Asco	Saprobic wood-degrading	Yes
*Trichoderma reesei*	Asco	Soft rot	Yes
*Thielavia terrestris*	Asco	Soft rot	Yes
*Daldinia eschscholzii*	Asco	Soft rot	Yes
*Chaetomium globosum*	Asco	Soft rot	Yes
*Chaetomium thermophilum*	Asco	Soft rot, soil	Yes
*Talaromyces leycettanus*	Asco	Soft rot, soil	Yes
*Penicillium decumbens*	Asco	Wood-degrading	Yes
*Serpula lacrymans*	Basidio	Brown rot	Yes
*Postia placenta*	Basidio	Brown rot	Yes
*Piriformospora indica*	Basidio	Endophyte	Yes
*Coprinopsis cinerea*	Basidio	Saprophyte	Yes
*Schizophyllum commune*	Basidio	White rot	Yes
*Trametes versicolor*	Basidio	White rot	Yes
*Lentinus polychrous cut500*	Basidio	White rot	Yes
*Pycnoporus cinnabarinus*	Basidio	White rot	Yes
*Phanerochaete chrysosporium*	*Basidio*	*White rot*	*Yes*

### Gene mapping

The genomes of the 39 fungi were screened with Hotpep for members of the GH families 1–131 and for the AA families 9–11 that include the LPMOs. The N_50_ for the genomes varied between 6,000 and 10,000 kilobase pairs (kbp) indicating different quality of the genome assemblies. However, gene mining with Hotpep was relatively insensitive to the contig lengths; e.g.; an initial sequencing of the genome of *Pycnoporus cinnabarinus* could only be assembled to an N_50_ of 3.4 kbp. Repetition of the sequencing gave a higher quality sequence that was assembled to an N_50_ of 96 kbp (Table S1). Nevertheless, Hotpep was able to find the same genes in the poor assembly as in the good assembly provided that all contigs shorter than 500 bp were discarded.

**Figure 2:**
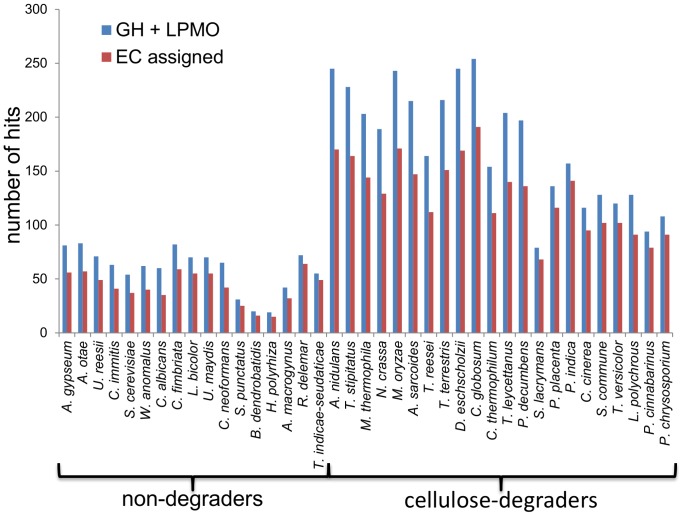
Number of GH- and LPMO-encoding genes and functionally predicted genes in the 39 fungi. All GH and LPMOs (CAZy auxiliary activity families AA9, AA10 and AA11) were annotated in the genome sequences of the 39 fungi. Next, as many as possible of these genes were assigned an EC number as described in “Materials and Methods”.

On average there were 174±53 GHs and LPMOs in the cellulose-degraders and 59±19 GHs and LPMOs in the non-degraders (Table S5). Interestingly, only 78 GHs and 1 LPMO were found in the dry rot fungus *Serpula lacrymans,* which was the only cellulose-degrading fungus with less GHs and LPMOs than some of the fungi classified as non-degraders (Fig. 2).

Of the 4823 GH- and LPMO-encoding genes that were found in the 39 fungal genomes 3544 could be assigned a function by Hotpep (Table S6) as they belonged to subfamilies including proteins with known enzymatic activities as previously described [43]. The remaining 17% of the GH-encoding genes were classified into subfamilies consisting of proteins without any described function and hence, could not be assigned a function. There was no difference (*P* = 0.50, Student's unpaired T-test) between the fraction of GHs that were assigned a function for the cellulose-degraders and for the non-degraders.

**Figure 3:**
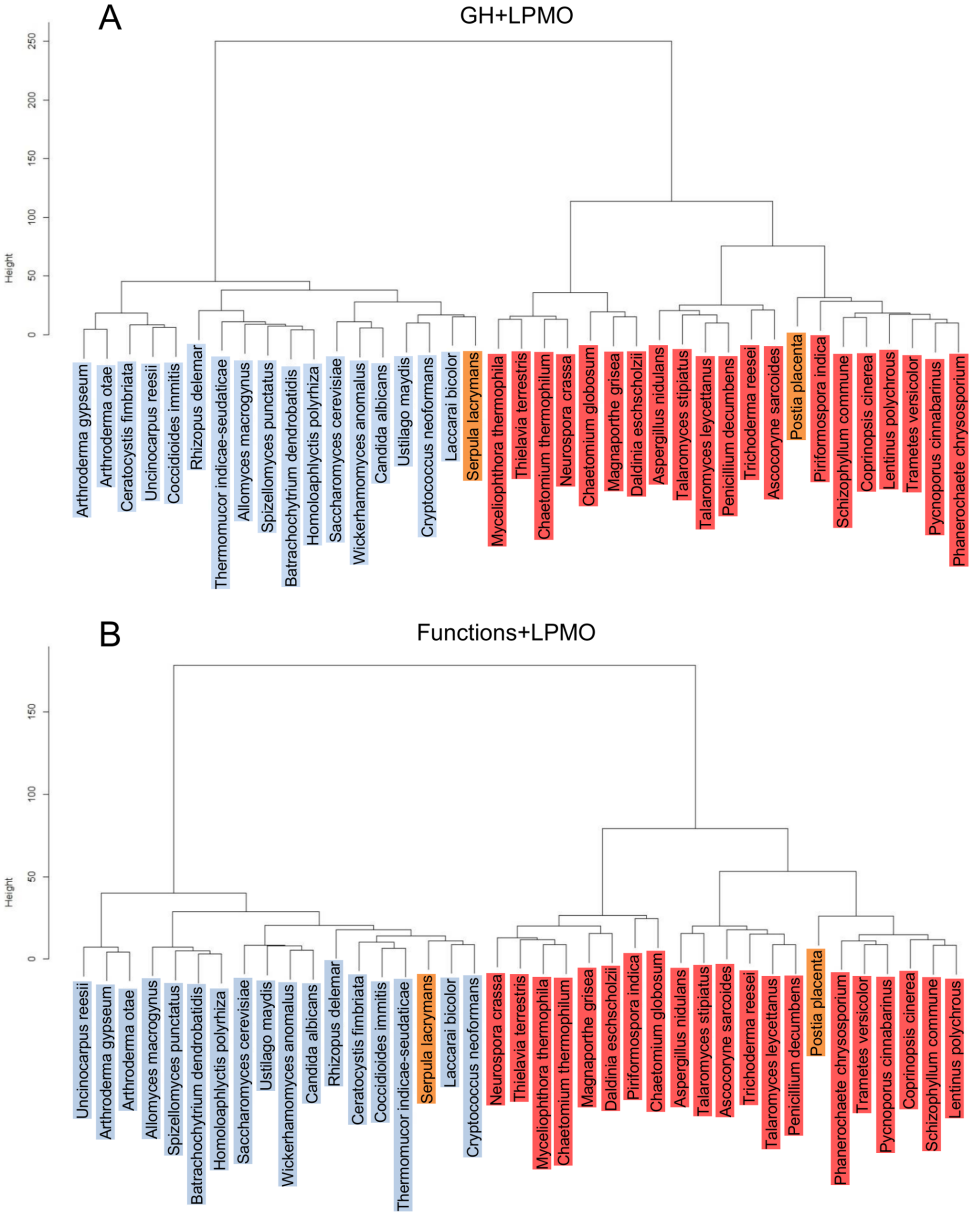
Cluster analysis based on GH- and LPMO-encoding genes of the cellulose-degrading and non-cellulose-degrading fungi. A) Cluster analysis based on the number and types of GH-families and AA families 9–11. B) Cluster analysis based on EC numbers for the GH genes an on the AA families 9 and 11. Cluster analysis was performed as described in “Materials and Methods”. Light blue is used as background color for non-cellulose-degraders, brown for brown rot and light red for other cellulose-degraders.

Cluster analysis based on GHs and LPMOs divided the fungi into two groups in agreement with the division of the fungi into cellulose-degraders and non-degraders except for *S. lacrymans* that clustered together with the non-degraders (Fig. 3a). This division implies that based on GHs and LPMOs the cellulose-degrading *Ascomycota* are more related to the cellulose-degrading *Basidiomycota* than to non-degrading *Ascomycota*.

Cluster analysis based on functions of the Hotpep-predicted functions of the proteins and assuming that all AA9 and AA11 family proteins are LPMOs [20], [21] gave the same two groups of cellulose-degraders and non-degraders (Fig. 3b). Moreover, the fungi clustered into the same two groups even when only the data for the cellulose-acting enzymes endoglucanase (EC 3.2.1.4), β-glucosidase (EC 3.2.1.21), cellobiohydrolases (EC 3.2.1.91 and EC 3.2.1.176) and the LPMOs were used for clustering (Fig. S1).

**Figure 4:**
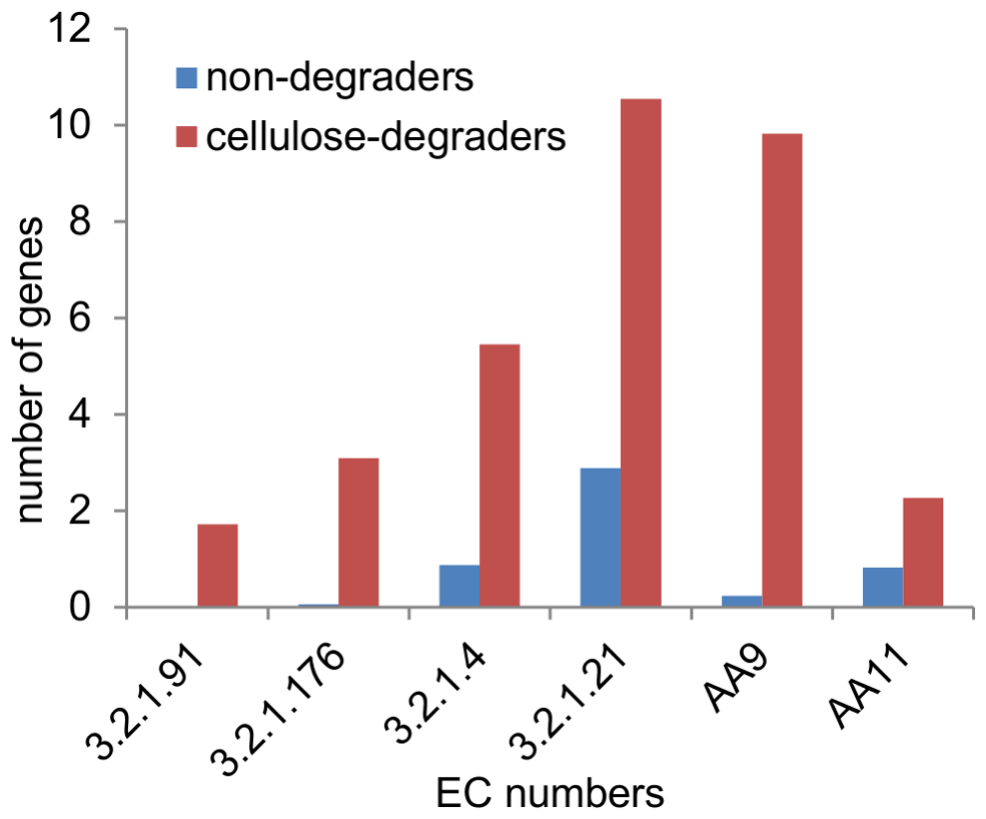
Average number of genes encoding each cellulose-degrading enzyme in the fungi. The average number of genes predicted to encode each of the activities endoglucanase (EC 3.2.1.4), β-glucosidase (EC 3.2.1.21) and cellobiohydrolases (EC 3.2.1.91, non-reducing end and EC 3.2.1.176, reducing end) and the LPMOs (AA9 and AA11) in the cellulose-degraders and non-cellulose degraders.

Interestingly, all of the cellulose-degraders except for the two brown rot fungi, *Serpula lacrymans* and *Postia placenta* have one or a few of each type of cellobiohydrolase whereas no cellobiohydrolase genes were found in the genomes of the 17 non-cellulose degraders (Fig. 4) except for a single CBHI in *Ceratocystis fibriata* (Table S6). A similar pattern was observed for the AA9-type LPMOs whereas several genes encoding endoglucanase, β-glucosidase and AA11-type LPMOs were found in the non-degraders (Fig. 4, Table S6). This difference may indicate that cellobiohydrolases and AA9 LPMOs are hallmarks for dedicated cellulose-degrading fungi that do not use Fenton chemistry. Another interesting finding is that on average each genome of the cellulose-degraders only had 2–3 genes encoding each type of cellobiohydrolase but 6–11 genes encoding AA9s, endoglucanases or β-glucosidases (Fig. 4). *Talaromyces stipitatus, Talaromyces leycettanus, Penicillium decumbens* and *Trichoderma reesei* were exceptions to this pattern as they only had 1–3 genes encoding AA9s in their genomes. Nevertheless, this pattern of gene copy number suggests that few types of cellobiohydrolases are necessary for cellulose degradation whereas several different enzymes with LPMO, endoglucanase and β-glucosidase activity are required.

In line with this notion, far fewer genes encoding cellulose-degrading enzymes were found in the dedicated white rot fungi *Trametes versicolor*, *P. cinnabarinus*, *Phanerochaete chrysosporium* and *Lentinus polychrous* than in some of the saphrophytic *Basidiomycota* and *Ascomycota* that can degrade many different types of plant biomass in nature (Table S6).

**Figure 5:**
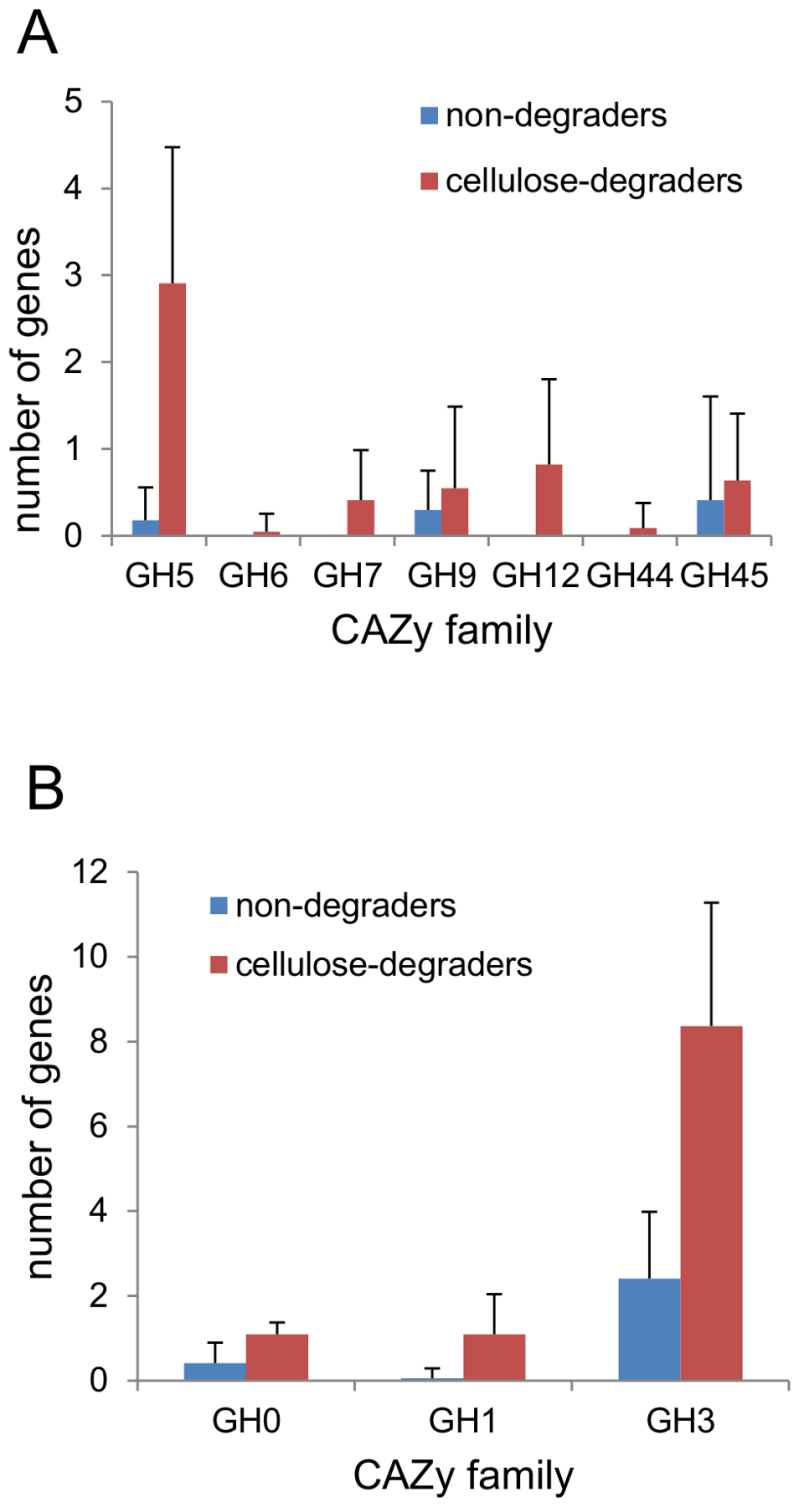
Distribution of endoglucanases and β-glucosidases in many different GH families. A) Average number of endoglucanase-genes in each GH families per fungal genome. B) Average number of β-glucosidase-genes in GH families per fungal genome. The GH families of each functionally annotated endoglucanase (EC 3.2.1.4) and β-glucosidase (EC 3.2.1.21) was counted.

Furthermore, the endocellulases of the cellulose-degraders were distributed in several GH families and the β-glucosidases were mainly from family GH3 but also from GH1 or unclassified (Fig. 5). Whereas these enzymes show large diversity, the cellobiohydrolases all belonged to either GH6 (CBHII) or GH7 (CBHI). The AA9-type LPMO-encoding genes found in the genomes of the cellulose-degrading fungi belonged to 12 different AA9 subfamilies (Fig. S2) in agreement with a relatively large sequence-diversity of these enzymes.

In contrast to LPMOs of the AA9 family, occurrence of genes encoding AA11-family LPMOs, originally described as a subfamily of the GH61s [22] did not correlate to cellulose-degradation. Instead, AA11 was found in all *Ascomycota,* except the yeasts *Saccharomyces cerevisiae* and *Wickerhamomyces anomalus,* and in the two *Basidiomycota Schizophyllum commune* and *Cryptococcus neoformans*. Furthermore, no AA10-type LPMOs were found.

**Figure 6:**
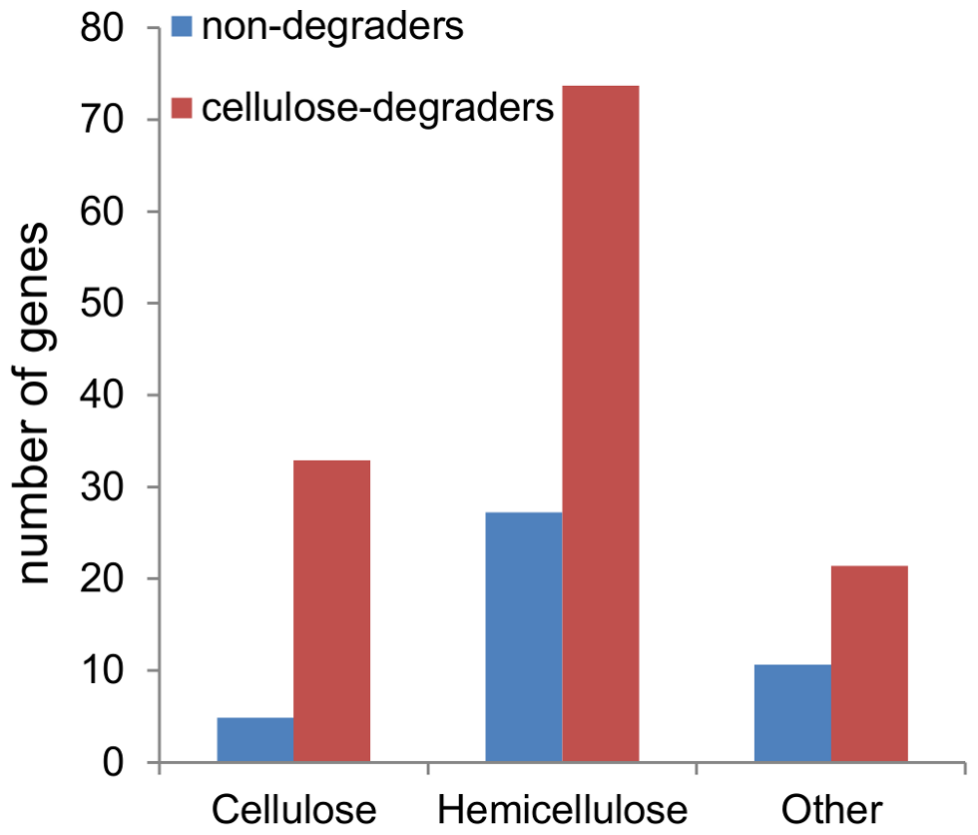
Average number of genes encoding glycoside hydrolase and LPMO enzymes in the 39 fungi. The average number of genes predicted to encode cellulose-degrading activities (EC 3.2.1.4, 3.2.1.21, EC 3.2.1.91 and EC 3.2.1.176 and LPMOs), hemicellulose-degrading activities (EC 3.2.1.37, 3.2.1.151, 3.2.1.8, 3.2.1.55, 3.2.1.23, 3.2.1.59, 3.2.1.131, 3.2.1.177, 3.2.1.78, 3.2.1.67, 2.4.1.183, 3.2.1.25, 3.2.1.31, 3.2.1.15, 3.2.1.39, 3.2.1.58, 3.2.1.63, 3.2.1.22, 3.2.1.99, 3.2.1.75, 3.2.1.52, 3.2.1.6, 3.2.1.106, 3.2.1.51, 3.2.1.113, 2.4.1.18, 2.4.1.25, 3.2.1.28, 3.2.1.45, 3.2.1.18, 3.2.1.3, 3.2.1.26, 3.2.1.24, 2.4.1.20, 3.2.1.40 and 3.2.1.171) or other activities (EC 3.2.1.1, 3.2.1.20, 3.2.1.10, 3.2.1.165, 3.2.1.132, 3.2.1.14, 3.2.1.145, 3.2.1.89 and 3.2.1.164) in the cellulose-degraders and non-cellulose degraders. The division of enzyme activities (EC numbers) on cellulose-degradation, hemicellulose-degradation or other was described previously [2].

Several of the gene families encoding hemicellulose-degrading enzymes such as xylan 1,4-beta-xylosidase, xyloglucan-specific endo-beta-1,4-glucanase, endo-1,4-beta-xylanase and alpha-N-arabinofuranosidase showed the same pattern of occurrence as the cellulases with one or more genes in almost all the cellulose-degraders, fewest in the brown rot fungi, and no genes in most of the non-degraders (Fig. 6, Table S6). In contrast genes encoding enzymes such as α-amylase or chitinase that attack other types of carbohydrate macrostructures followed a different pattern of occurrence.

## Discussion

The genomic analysis of GH- and LPMO-encoding genes in 39 fungi identified a set of cellulose-degrading enzyme activities found in almost all the fungi capable of decomposing the cellulose found in plant cell walls in nature [4], [5], [28], [40]. These activities include endoglucanase (EC 3.2.1.4), β-glucosidase (EC 3.2.1.21), cellobiohydrolases CBHI (EC 3.2.1.176) and CBHII (EC 3.2.1.91) and the AA9-type LPMOs. This finding is in agreement with current models for degradation of crystalline cellulose that suggest that cellulose is decomposed by a combined action of solubilization by CBHI and LPMOs and disentanglement by CBHII and endocellulases [2], [6], [7].

Curiously, with the exception of *Ceratocystis fimbriata*, CBH- and AA9-type LPMO-encoding genes were only present in the cellulose-degraders whereas genes encoding endoglucanases and β-glucosidases were found in many of the non-degraders. Many endoglucanases and β-glucosidases are enzymes that can act on solubilized cellulose and β-1, 4-glucose oligomers (For review see [2]). Hence, finding these enzymes in non-cellulose degraders supports the notion that some fungi that are not dedicated plant cell-wall degraders are able to degrade easily accessible cellulose such as pure cellulose not embedded in plant cell walls or soluble β-glucans, anyhow. The two *Zygomycetes T. indicae-seudaticae* and *R. delemar* are typical examples of fungi that grown on easily accessible substrates [41] but nevertheless secrete cellulases and *T. indicae-seudaticae* is even able to grow on crystalline cellulose [43].

An interesting observation was that, on average, the cellulose-degrading fungi had two CBHII and three CBHI genes but six endoglucanase genes, ten AA9 genes and eleven β-glucosidase genes in the genome. Different endoglucanases and β-glucosidases may have properties that make them suitable for degradation of cellulose in different plant cell wall materials and under different conditions. For example, β-glucosidases with high activity against cellobiose may also be more prone to product inhibition [46] and it was shown that one of the many β-glucosidases from *Aspergillus niger* has a special structure that may be useful for preventing unproductive binding of cellulases to lignin thereby enhancing hydrolysis of cellulose in a complex substrate [47]. It has also been reported that different endoglucanases have different capabilities for synergistic interaction with cellobiohydrolases [48] and that the presence or absence of a carbohydrate-binding module can drastically affect the properties of endoglucanases [49]. Hence, it is clear that enzymes with the same activity can have different properties that make them suitable for participating in cellulose-degradation in different contexts.

Less is known about the enzyme activities of LPMOs but the genome of the cellulose-degrading fungus *Chaetomium globosum* has at least 33 genes encoding putative LPMOs [50]; 35 genes were found in the present study. Such a vast number of enzymes with the same function seems to be excessive for the degradation of pure cellulose but is relevant in the context of the natural cellulose-containing substrates of *C. globosum*. Thus, it is likely that the reason some fungi have several AA9-encoding genes in their genomes is the same as for the endoglucanases and the β-glucosidases: Namely to be able to decompose different substrates. Interestingly, recent reports show that some AA9 LPMOs are able to degrade soluble glucans [51] and xyloglucans [52].

The host of different plant cell wall-degrading enzymes with the same enzymatic activity found in each fungal genome indicates that a specific enzyme composition is necessary for decomposition of each fungal substrate. One interesting implication of this finding is that it might be possible to optimize the composition of enzyme blends for industrial degradation of different types of lignocellulosic biomass.

The occurrence of AA11-type LPMOs did not seem to correlate with cellulose-degradation in agreement with that the only AA11 that has been enzymatically characterized is a chitin-active LPMO [21].

The only cellulose-degraders that did not possess genes for both CBHI and CBHII cellobiohydrolases were the two brown rot Basidiomycetous fungi *Serpula lacrymans* and *Postia placenta* that use Fenton chemistry for cellulose degradation [45], [53], [54]. These fungi also had very few LPMO genes. It has been suggested that the brown rot fungi have undergone the same gene loss during their evolution as ectomycorrhizal fungi such as *Laccaria bicolor* [45], [55] and *Paxillus involutus* [56] and hence, when the Fenton-chemistry was acquired they may have lost their cellobiohydrolase genes as the exocellulase activity became superfluous. However, it was recently reported that the brown rot fungus *Coniophora puteana* has two genes from each of the GH6 and GH7 families that encode the cellobiohydrolases and that fungi that have a wood decay mode placed in between brown rot and white rot have the same cellulolytic enzymes as the white rot fungi [28]. Hence, the most important distinction of the brown rot fungi may be the lack of ligninolytic class II peroxidases [28].

The relatively few genes encoding cellulose-degrading enzymes in the dedicated white rot fungi suggest that the number of genes encoding cellulose-degrading enzymes in a fungal genome is related to the diversity of the substrates that the fungus can utilize rather than to the efficiency with which the fungus degrades the substrate. In this context it is interesting that different fungi participate in different parts of the degradation of leaves in nature although this substrate contains cellulose throughout the decomposition and both endoglucanase and β-glucanase activity is detectable at almost all time points [57]. The enzymes expressed by the fungi acting at the beginning of leaf-degradation are likely to encounter a more complex substrate, which require a more diverse arsenal of enzymes, than the fungi acting in the final stages of leaf-degradation. Similar fluctuations in population diversity of fungi has been reported for degradation of spruce [58].

Five of the *Ascomycetes* analyzed, *Talaromyces stipitatus, Talaromyces leycettanus, Penicillium decumbens, Trichoderma reesei*, and *Ascocoryne sarcoides*, only had few AA9 LPMO genes although they contained many endoglucanase- and β-glucosidase-encoding genes. This result suggests that these fungi may occupy a different ecological niche than the other cellulose-degrading *Ascomycetes* or that they may use a different mechanism to degrade cellulose.

In agreement with their ability to degrade cellulose the genomes of the cellulose-degraders also encode more glycoside hydrolase activities not directly used for cellulose degradation than the non-degraders. This correlation is in agreement with that many of these fungi have been described as degraders of non-cellulose components of the plant cell wall like hemicellulose and pectin through the action of glycoside hydrolases [2]–[5]. However, as expected from their substrate specialization far fewer genes encoding non-cellulolytic glycoside hydrolases were found in the genomes of the white and brown rot fungi than in the saprophytic fungi.

Clustering of the fungi based on the type and number of all GH- and LPMO-enzymatic activities divided the fungi into the anticipated groups of cellulose-degraders and non-degraders with the exception of *S. lacrymans,* which degrades cellulose by a unique Fenton chemistry-based method that does not require external moisture [45], [53], [54]. This fungus grouped together with the non-degrading ectomycorrhizal fungus *L. bicolor*. The clustering of cellulose-degrading species of *Ascomycota* and *Basidiomycota* in the same group rather than together with the taxonomically more related non-cellulose-degrading fungi indicates that although *Ascomycota* and *Basidiomycota* diverged between 400 and 1,800 Mya [1], [59], these fungal divisions have evolved the same enzymatic solutions for degradation of the cellulose in plant cell wall material. Hence, based on GHs and LPMOs the cellulose-degrading *Ascomycota* are more related to cellulose-degrading *Basidiomycota* than to non-degrading *Ascomycota*.

This study confirms that PPR can be used for subdividing glycoside hydrolase families and that the resulting subfamilies correlate to a large extent to the function of the proteins [22]. It further documents that prediction of function from sequence is a powerful approach to address important biological questions about fungal physiology and nutrition by characterizing the molecular development and relatedness of the fungal lignocellulose-metabolic secretome. In the present work we found that the method could be used as a fast and automatic approach not only to predict the function of known glycoside hydrolases but also to annotate all glycoside hydrolases and LPMOs in a fungal genome and to predict their function. The functional annotation is an important feature of PPR and Hotpep as it provides a direct route from primary sequence to function.

## Supporting Information

Figure S1 **Cluster analysis based on the number of endoglucanase, β-glucosidase, cellobiohydrolases and LPMO-encoding genes.** Cluster analysis was performed as described in “Materials and Methods”.
(TIF)

Figure S2 **Distribution of AA9-encoding genes in AA9 subfamilies.** The AA9 subfamily generated by PPR of each AA9 was counted.
(TIF)

Table S1 **List of fungi, assembly number and N50 of the assembly**
(XLSX)

Table S2 **Key assembly data for the genomes sequenced in the present work.**
(XLSX)

Table S3 **Comparison of number of GH and AA9-11 genes found in each of six fungi by PPR to the data in CAZy.**
(XLSX)

Table S4 **Comparison of number of genes found in each GH and AA9-11 family by PPR to the data in CAZy.**
(XLSX)

Table S5 **Number of GH and AA9-11-encoding genes found by Hotpep in each fungus.**
(XLSX)

Table S6 **Functional annotation of GH and AA9-11-encoding genes in each fungus.**
(XLSX)
