# Supplementary figures and images for: Several Genes Encoding Enzymes with the Same Activity Are Necessary for Aerobic Fungal Degradation of Cellulose in Nature
Source: PLoS One. 2014 Dec 2;9(12):e114138. doi: 10.1371/journal.pone.0114138 (PMC4252092; doi:10.1371/journal.pone.0114138)

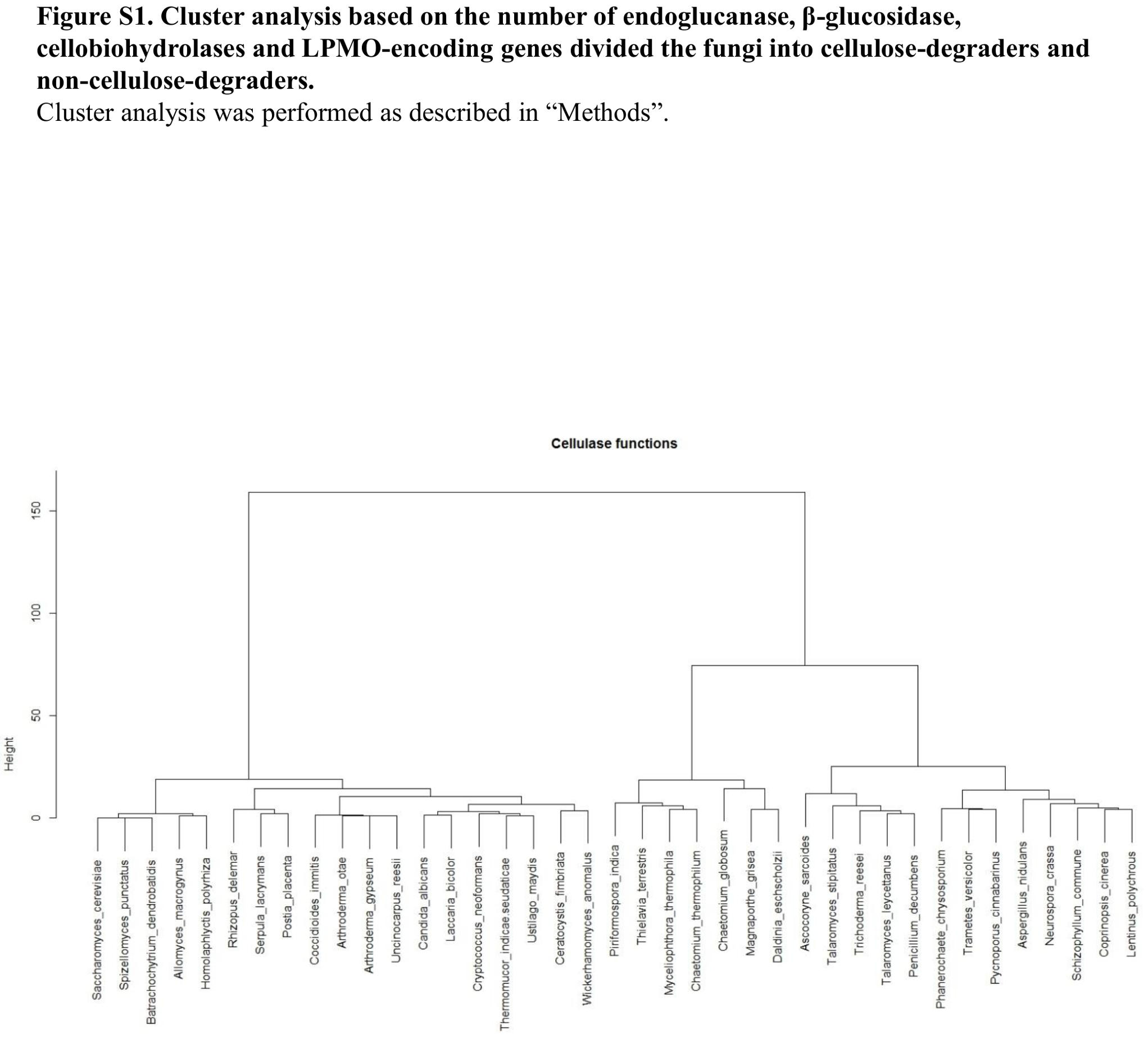

Supplement: Figure S1 — Cluster analysis based on the number of endoglucanase, β-glucosidase, cellobiohydrolases and LPMO-encoding genes. Cluster analysis was performed as described in “Materials and Methods”. (TIF) [file pone.0114138.s001.tif]

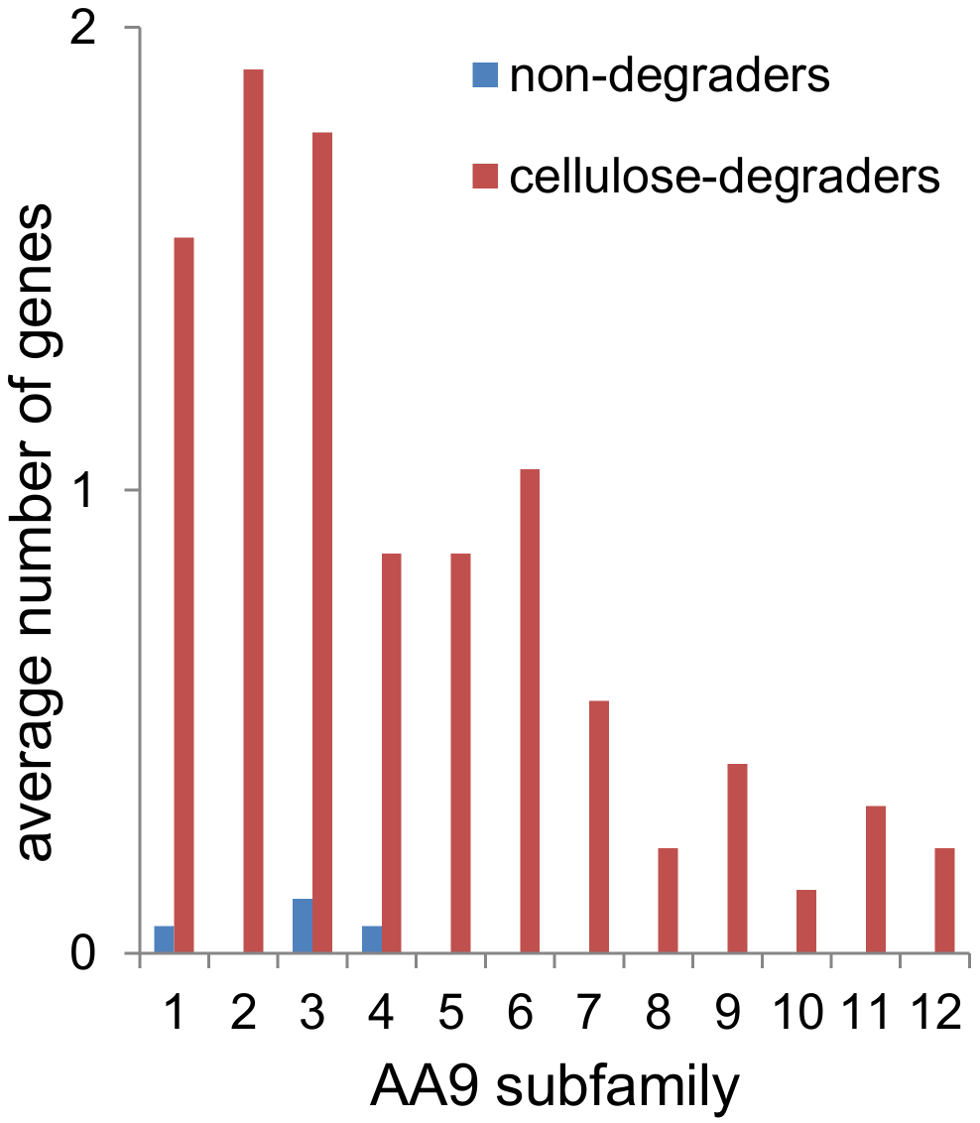

Supplement: Figure S2 — Distribution of AA9-encoding genes in AA9 subfamilies. The AA9 subfamily generated by PPR of each AA9 was counted. (TIF) [file pone.0114138.s002.tif]
